# The Impact of Progesterone and Estrogen on the Tooth Mobility

**DOI:** 10.3390/medicina59020258

**Published:** 2023-01-29

**Authors:** Małgorzata Peruga, Joanna Piwnik, Joanna Lis

**Affiliations:** 1 Individual Medical Practice, 93-410 Łódź, Poland; 2 Physics and Applied Informatics, 92-321 Łódź, Poland; 3 Adult Orthodontics Clinic, Department of Dentofacial Orthopedics and Orthodontics, Wroclaw Medical University, 50-376 Wrocław, Poland

**Keywords:** progesterone, estrogen, hormonal contraceptives, periodontal ligaments, bone metabolism, tooth mobility

## Abstract

*Background and Objectives*: Progesterone and estrogen modify the bone metabolism directly related to the periodontium, this study aimed at answering the question whether fluctuations in the levels of these hormones or the use of their synthetic equivalents in modern contraceptives have a significant impact on the natural tooth mobility (TM) in its alveolus. *Materials and Methods*: Sixty healthy women who had never been pregnant and when interviewed reported either (1) having regular menstruations every 28–30 days or (2) taking oral two-phase two-ingredient hormonal contraceptives formed, respectively, groups M and S in the study. TM evaluated as the Periotest value (PTV) was checked in the menstruation, ovulation, and luteal phases of the menstrual cycle (group M) and on the days corresponding to the moment of the menstrual cycle in group S. *Results*: Although the PTV-s were within the limits of norm, the canines and the molars were always more stable than the other teeth. In group M, the TM was statistically comparable (*p* > 0.05) in the menstrual and ovulation phases, thus significantly increased (*p* < 0.001) in the luteal phase. The TM remained constant (*p* = 0.758) in all studies in group S. The results demonstrated that the canines and the molars in the luteal phase were significantly more mobile in group M than in group S (*p* < 0.001), although increased mobility of the teeth in group M affected the canines and the first molars to a significantly lesser degree than the other teeth. *Conclusions*: However, since women between 20 and 30 years old constitute the majority of ortho-dontic patients, possible determination of the optimum moment of force application in relation to the sex hormones cycle, namely, to its luteal phase, is clinically very promising.

## 1. Introduction

The female menstrual cycle is a sequence of recurrent hormonal fluctuations, primarily of estrogen and progesterone. Levels of these hormones differ in various periods of women’s lives: adolescence, menstruation, pregnancy, menopause, or when taking hormonal contraceptives [1,2].

Estradiol is an estrogen produced by female gonads, placenta, and some peripheral tissues (mainly adipocytes). It plays a key role in the development of secondary sex characteristics, and also contributes to the development of the limb and axial skeleton [3,4,5]. The latter effect is of key importance for orthodontists and periodontologists, as the periodontium is made of—among others—the cortical plate of the alveolus with an adjoining scaffolding of cancellous bone trabeculae. Estrogen and progesterone receptors are also located in other periodontal components, namely, the gingiva and the periodontal ligaments [6,7], so it has already been proven that estrogen exerts a range of effects on tissues in the periodontium [8,9,10]. By regulating the production of epithelial glycogen, it reduces the keratinization of the gingiva, ultimately decreasing the role of the epithelial barrier. It reduces the reaction of T-lymphocytes, thus leading to an increased occurrence of gingival inflammations, without favoring dental plaque formation. Estrogen also stimulates the proliferation of fibroblasts and the synthesis and maturation of tissues composing the gingiva. It reduces the production of leucocytes in bone marrow, impeding the release of pro-inflammatory cytokines by human marrow cells [11,12].

Progesterone is released by the corpus luteum, placenta, and adrenal cortex. The literature on orthopedics presents an extensive body of information on its impact on bone metabolism and the effects on ligaments. It expands vascular beds, increasing their permeability. It stimulates the production of prostaglandins and contributes to increased levels of multinuclear leucocytes, in contrast to estrogen [2]. Progesterone impedes the synthesis of collagen present in the periodontal ligaments. As a result, their potential to repair reduces, as does the number of their fibers. An increase in the level of progesterone accelerates the metabolic decomposition of folic acid required for growth, development, and proliferation of the cells. Progesterone levels are the highest during the luteal phase of the menstrual cycle, namely, 7 days after ovulation, whereas estrogen levels are the highest during ovulation [1,2,10].

The natural cyclicality of fluctuations of the discussed hormones is disrupted by using hormonal contraceptives, the effects of which include stopping ovulation. Women who have not given birth are usually prescribed two-ingredient two-phase pills, which contain ethinylestradiol and synthetic equivalents of progesterone–gestagen. These pills change the level of estrogen and gestagen throughout the entire menstrual cycle, in contrast to single-phase pills [12,13,14,15,16]. Current contraceptives contain much lower doses of hormones than pills used in the past. Nevertheless, it has been proven that modern contraceptives may still significantly modify the immune response, resulting in a risk of damage to the periodontium due to an increased number of anaerobic bacteria in comparison to aerobic bacteria and the increased prevalence of tooth decay [16,17,18,19,20,21].

Given the fact that progesterone and estrogen modify the bone metabolism directly related to the periodontium, a question arises whether fluctuations in the levels of these hormones or the use of synthetic equivalents in modern contraceptives have a significant impact on the natural mobility of the teeth in their alveoli.

### Objective of the Study

The objective of the study was to assess the profile of the tooth’s natural mobility in the alveolar processes during the menstrual cycle and while taking hormonal contraceptives.

## 2. Materials and Methods

### 2.1. Materials

**Figure 1:**
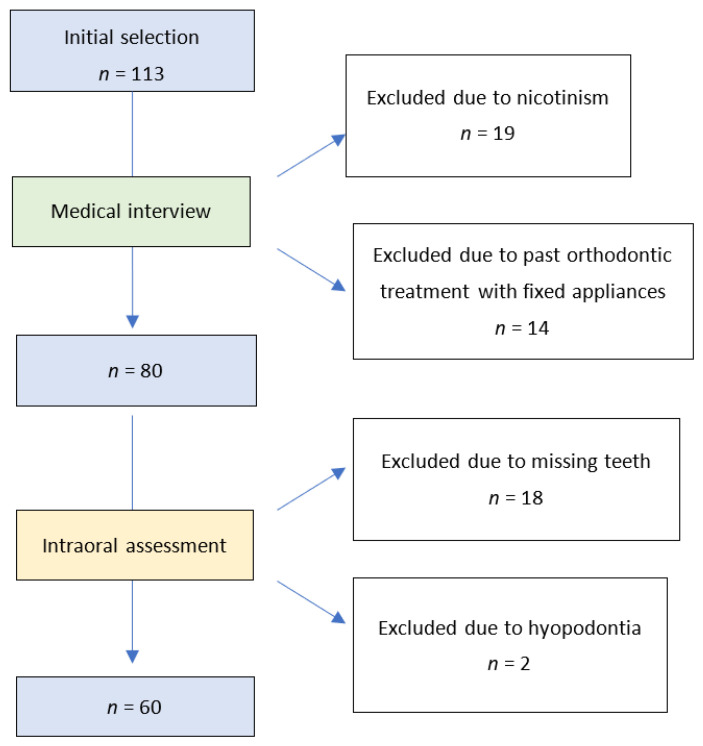
Flowchart of the study material selection.

Participants in the study were chosen among women in good general health, with a normal body mass index (18.5–24.9), who reported for an orthodontic consultation to the individual medical practice, had never been pregnant, and when interviewed reported either (1) having regular menstruations every 28–30 days or (2) taking oral two-phase two-ingredient hormonal contraceptives for at least four months, which was the factor deciding which group a given participant was assigned to. With regard to oral health, the inclusion criterion was the lack of both the chronic inflammations in the oral cavity and the active caries lesions. A total of 113 women were initially enrolled in the study; the process of further selection based on the application of exclusion criteria is shown on the flowchart (Figure 1).

All women that required conservative, periodontal, or surgical treatment, as determined during an initial examination, received appropriate referrals to dental specialists. Women with irregular menstruations were referred to a gynecologist.

Ultimately, 60 women between 20 to 30 years old qualified for the prospective study. Written informed consent for testing was obtained from all participants; the tests took place between January and April 2019.

### 2.2. Methods

Chosen women were divided into two groups that underwent a series of three examinations (Table 1). Each participant was instructed to report for testing on strictly specified days and at a specific, constant time.

The examination was designed to enable achieving the goal of our study. For this purpose, a single sample of venous blood was always taken at 7.00 am, each time from the cubital fossa to assess the axis of the hypothalamus–pituitary gland–ovary and to determine the blood levels of progesterone and estrogen subsequently evaluated by a gynecologist. The tooth mobility was assessed objectively using a Periotest electrical device that measures the deviation and reduction in speed of a small headpiece (that comes out from inside the machine) while it hits a tooth in its alveolus. Contact time is converted into numerical values of the Periotest, referred to as PTV (Periotest value). The range of the Periotest values was −8 to +50. The higher the PTV, the higher the mobility of the tested object [22,23,24]. Three measurements were taken at each tooth, and the average result was recorded.

### 2.3. Statistical Analysis

The StatSoft Statistica 13.3 software package was used to perform a statistical analysis of the intra- and intergroup comparisons of the clinical parameters. The significance level was set to α = 0.05. The Shapiro–Wilk test was used to examine the normality of the data distribution. As the data distribution was not normal, the results were presented as a median (interquartile range, IQR). The Kruskal–Wallis non-parametric test was used to compare the tooth mobilities within groups M and S and the Mann–Whitney U test was used to compare the mobility of each tooth between groups M and S. Median ranges of all group pairs were compared in order to perform a post-hoc analysis. Results of the post-hoc analysis were evaluated using the Mann–Whitney U test. The mobility of the teeth in group M was compared using Friedman’s ANOVA non-parametric, and the Wilcoxon test was twice used for post-hoc analysis. Mobility of the teeth in group S was compared using the Mann–Whitney U test, reducing the level of significance in multiple comparisons using the Bonferroni correction.

## 3. Results

Despite all of the PTV results being within the normal range, the results of the examinations from I to III demonstrated that the tooth mobility varied, namely, the median PTV values in both groups differed statistically (Table 2). Moreover, the canines and the molars were always significantly more stable than the other teeth (Table 3). Significantly lower mobility of the canines and the molars in comparison with the other teeth was independent of both the day of the menstrual cycle and the duration of using contraceptives.

In women who menstruated (group M), the tooth mobility was statistically comparable (*p* > 0.05) in examinations I and II (i.e., during the menstrual phase and during ovulation), however, increased significantly (*p* < 0.001) in examination III (i.e., in the luteal phase of the menstrual cycle). The tooth mobility remained constant (*p* = 0.758) in all examinations in group S, irrespective of the duration of medication (Table 4). This mobility was significantly higher in women from group M than in women from group S. The highest difference occurred in examination III, and therefore it underwent additional comparative analysis. For its purpose, homonymous teeth in both quadrants were treated as one, which resulted in a lower number of multiple comparisons (Table 5). The results demonstrate that the canines and the molars in examination III were significantly more mobile in group M than in group S (*p* < 0.001), although increased mobility of the teeth in group M affected the canines and the first molars to a significantly lesser degree than the other teeth.

## 4. Discussion

During the menstrual cycle in healthy women in their procreative period, the level of progesterone rises until the seventh day after the egg cell is released from the ovary, and then—if fertilization occurs—continues to occur in later stages of the pregnancy [1]. Without fertilization, the levels of progesterone in the patient’s blood drop, resulting in menstruation in further cascade reaction. In healthy women, progesterone levels should range between 0.057 and 0.893 ng/mL during the follicular case, between 0.121 and 12.0 ng/mL during the ovulation phase, and between 1.83 and 23.9 ng/mL during the luteal phase. In post-menopausal women, these levels should range from 0.05 to 0.126 ng/mL [1,2]. To ensure that the values have a point of reference in any study, the study must include women from a homogenous group, which was the case in our study. Participants in our study were women of similar ages, from whom we sampled blood at a constant set time, within a short period of time (January–April). In this way, we were able to rule out the impact of factors such as age, time of day, and season on the result of the test. By referring to numerical data [1], which indicated the level of progesterone in the luteal phase increases by nearly four times in comparison to the follicular phase, the PTVs we obtained in study III in group M enabled us to identify this hormone as the driving force behind the increased average mobility of teeth.

Choosing Periotest as a measurement instrument was also fully justified. Periotest is widely accepted and used to measure mobility in vivo and in vitro in periodontology, implantology, orthodontics, and traumatology. The advantages of the Periotest include its ease of use, ability to make measurements in the horizontal and in the vertical dimensions, and primarily the repeatability of results [22,25]. Thus, our studies can be reproduced at any scientific center or even a medical practice.

As the PTVs measured in our tests ranged between −8 and +9 [26], which is considered proof of stability of a tooth, we were able to demonstrate that changes in the female menstrual cycle caused by contraceptives do not have a destructive effect on young and healthy periodontium.

However, this does not mean that there is no link between the movement of teeth and contraceptives. On the contrary: the long-term use of hormonal pills has a stabilizing effect on the anchoring of the teeth in the alveolus, as the tooth mobility among women who took contraceptives (group S) was higher than in menstruating women (group M) only in examination I. In further examinations, tooth mobility was greater in group M than in group S, although a statistically significant inter-group difference was observed only in examination III.

The use of hormonal therapies is an important issue for both orthopedists and dentists, as it may result in disruptions to the physiological development of bone and its density [1]. Unfortunately, there too few studies have been conducted on adolescent women, and those that involved young and mature women not only did not formulate clear conclusions on the effects of taking hormonal contraceptives, but also had divergent results [27,28], and as such offer little value as evidence. However, the fact that estrogen promotes the termination of bone growth including the mandible has been demonstrated beyond doubt [29,30,31,32,33], and therefore the effects of this hormone may affect the tooth mobility.

Since the metabolism of the alveolar ridge cannot be considered the cause for the changes in PTV among women who menstruate and women who take hormonal contraceptives, we needed to focus on the second component of the periodontium, namely, the periodontal ligaments, particularly as the literature indicates that hormonal contraception leads to an increased risk of breaking the attachments of ligaments in the knee joint [34,35,36,37]. In this aspect, our results are hugely significant. Without animal testing, we were able to demonstrate that the impact of progesterone and estrogen on the periodontium was different to that on the rest of the human system: we did not observe any reduction in the function of periodontal fibers in the representative, homogenous group S.

Hyperplasia of the gingiva—another part of the periodontium—is a documented phenomenon in healthy women who use hormonal contraceptives in the long-term [38,39,40]. Unfortunately, researchers have been unable to determine the limit dose of hormones that affects the above-described changes [41,42,43]. However, as sex hormones reduce the immune response of the gingiva to the dental plaque, hormonal contraception, in particular estrogen-based, may intensify the action of localized irritating factors [44,45], especially given that estrogen is responsible for keratosis of the gingiva and proliferative changes in its epithelium and increased fibroblast activity [1,2,10]. On the other hand, by increasing the permeability of microvessels, progesterone causes increased irritability and swelling of the gums and facilitates the resorption of bones and reduces collagen production, in this way promoting the catabolism of tissues and hampering their repair, and as such, may deepen gingival pockets, particularly with improper hygiene [1,2,10,41,42,43]. All of these processes contribute to the destabilization of the anchoring of teeth in the alveoli, but the obtained PTVs did not confirm the occurrence of this negative phenomenon.

The bacterial biofilm is another important area that was studied in terms of the effects of progesterone and estrogen on the movement of teeth. Bacterial biofilm is defined as a highly specialized, single- or multi-species form of microorganism life, permanently located on its substrate and surrounded by a layer of extracellular polysaccharides that create mucus [45]. Pioneers inhabiting the biofilm include strep bacteria: *Streptococcus mitis*, *Streptococcus oralis*, and *Streptococcus sanguis*. Other microorganisms include *Actinomyces* and Gram-negative bacteria (e.g., *Haemophilus*) [46]. They reduce the resting pH of the dental plaque [47,48,49], which reaches its lower values (pH = 4) near the maxillary incisors. This is associated with the free flow of saliva that contributes to the long-term retention of acids in the dental plaque as well as with the weight of saliva and distance between the incisors and the openings of salivary ducts [50]. Low pH leads to fast fluoride loss and reduces its cariostatic action, the limit point of which is pH = 4.5 [49,51]. *Streptococcus mutans* and *Lactobacillus* may reduce the pH of the dental plaque to <4.5, which is of huge importance, as Ali et al. [16] noted that the number of bacteria in women taking oral contraceptives was increased. The researchers stressed that the long-term use of hormonal contraceptives had a destructive effect on the delicate structures of the periodontium. Another significant fact related to these structures is that low pH contributes to an increase in the number of microorganisms from the *Candida* genus (i.e., *Porphyromonas gingivalis*, *Porphyromonas intermedia*, and *Actinobacillus actinomycetemcomitas*) in the gingival pockets [16]. As contraceptives are conducive to the proliferation of microorganisms, which cause the acidification of the oral cavity, which in turn poses a threat to the periodontium, increased mobility of teeth in the frontal section should be expected [16]. This was confirmed by the results of our studies, in which women who took contraceptive pills had less stable incisors than in women with a physiological menstrual cycle, in every phase.

With regard to the modern scope of our study, nowadays, oral contraceptives are safe and effective. The previous generation of medication contained much higher doses of synthetic hormones than those currently used and was based on a single ingredient, causing much more disruption [52]. Hormone doses had to be reduced due to their harmful effects on the cardiovascular system in large doses [1,2,10]. Unfortunately, despite the widespread and long-term use of oral contraception around the world, there is still no clear data on its impact on the condition of the bones of teenagers and young adult women available. Studied parts of the body (spinal bones, femoral necks, alveolar process), dates of studies (the 1980s and the early 21st century), their durations (from 1 year to 5 years), length of use of contraceptives and their types, and populations of studied patients (ethnic origin, age of 14 to 30, body mass, height) significantly varied. Therefore, interpretation of the results of all studies is extremely difficult. Studies undertaken on young women on short-term hormonal therapy in low doses did not show any changes in bone build and disorders in the periodontium. It has to be noted that selecting patients is quite difficult, as long-term studies of young adults taking hormonal contraceptives are often limited due to discontinuation or change of medication, and result in their hormonal composition. Nevertheless, these studies continue to create opinions among dentists, who are convinced that oral contraceptives increase the risk of gum and periodontal inflammation. It is worth noting that in order to determine whether modern oral contraceptives have any effect on the periodontium, much fewer studies have been conducted using modern medication than older generation medicines. Despite the incontrovertible significance of the results of our study on the impact of modern contraceptives on the periodontium during their use, we cannot lose sight of the observations of the long-term effects that are required from the scientific perspective and evidence-based medicine.

## 5. Conclusions

Given the fact that progesterone and estrogen modify the bone metabolism directly related to the periodontium, this study aimed at answering the question of whether fluctuations in the levels of these hormones or the use of their synthetic equivalents in modern contraceptives have a significant impact on the natural tooth mobility (TM) in its alveolus. Long-term use of hormonal pills has a stabilizing effect on the anchoring of teeth in the alveolus, as the tooth mobility among women who took contraceptives (group S) was higher than in menstruating women (group M) only in examination I. In further examinations, tooth mobility was greater in group M than in group S, although a statistically significant inter-group difference was observed only in examination III. Although the PTVs were within the limits of norm, the canines and the molars were always more stable than the other teeth. Although the exam involved a large group of women, the results of our research into the impact of hormonal contraception on PTVs are still merely preliminary findings.

However, since women between 20 and 30 years old constitute the majority of orthodontic patients, possible determination of the optimum moment of force application in relation to the sex hormones cycle, namely, to its luteal phase, is clinically very promising. Certainly, determining whether long-term intake of contraception may change the bone and periodontal ligament structures and whether such changes are reversible after the patient ceases to take medication, and if so, how quickly they reverse after medication is stopped, requires long-term studies.

## Figures and Tables

**Table 1:** Composition of the study material and timing of the examination.

	Examination		
	I	II	III
Women who menstruate, group M *n* = 30	Third day of the cycle Menstruation phase	Ovulation day Ovulation phase	7 days after ovulation Luteal phase
Women taking contraceptives, group S *n* = 30	1st day *	42nd day *	77th day *

* Day corresponding to the moment of the menstrual cycle in group M.

**Table 2:** Statistical analysis of the intra-group differences of PTV (obtained for the individual teeth).

	Group	Tooth	16	15	14	13	12	11	21	22	23	24	25	26	*p*
Examination I	M	Median (IQR)	0 (−0.3,0)	1 (0.4,2)	1 (0.3,2.8)	−0.1 (−0.2,0.1)	2.1 (1.5,3)	2.1 (1.2,2.8)	2 (1.2,2.7)	2 (1.4,3)	−0.1 (−0.2,0.1)	1 (0.3,2.8)	1 (0.4,2)	0.1 (0,0.5)	<0.001
	S		0 (0.4,0.4)	1 (0.3,2.1)	1 (0.5,2)	−0.1(−0.5,0.1)	2 (1.5,3.2)	1.8 (1.2,3.2)	1.9 (1.1,3.1)	2 (1.3,3)	−0.1 (−0.4,0)	1 (0.5,2)	1 (0.6,2.1)	0.2 (0,0.9)	<0.001
		*p*	0.40	0.89	0.58	0.60	0.97	0.86	0.72	0.84	0.14	0.58	0.21	0.33	
Examination II	M	Median (IQR)	0.2 (0,0.9)	1 (0.6,2.1)	1 (0.3,2.8)	−0.1 (−0.2,0.2)	2.1 (1.6,3)	2.1 (1.2,2.8)	2.1 (1.2,2.7)	2 (1.4,3)	−0.1 (−0.2,0.1)	1 (0.3,2.8)	1 (0.4,2)	0.4 (0.1,0.8)	<0.001
	S		−0.1 (−0.4,0.4)	1.1 (0.6,2.5)	0.9 (0.6,2.3)	−0.1 (−0.4,0.2)	2 (1.7,3.2)	2.1 (1.1,3.1)	1.7 (1.1,3)	1.9 (1.2,3)	−0.2 (−0.5,0)	1 (0.6,2.1)	1.3 (0.5,2.2)	0.2 (−0.4,0.8)	<0.001
		*p*	0.026	0.98	0.75	0.36	0.83	0.75	0.59	0.73	0.12	0.54	0.55	0.20	
Examination III	M	Median (IQR)	1.1 (0.5,1.9)	1.2 (0.6,2.3)	1.1 (0.4,3)	0.1 (0,0.4)	2.1 (1.6,3.1)	2.2 (1.3,3)	2.2 (1.3,2.8)	2.2 (1.5,3.1)	0 (0,0.2)	1 (0.4,3)	1.1 (0.6,2.1)	1.1 (0.8,1.9)	<0.001
	S		0.1 (−0.5,0.5)	1.1 (0.3,2.1)	1.3 (0.4,2.3)	−0.2 (−0.6,0.2)	2.2 (1.2,3.4)	2 (1.2,3.4)	1.9 (1.4,3.1)	2.2 (1.2,2.9)	−0.3 (−0.6,0.1)	0.9 (0.3,2.4)	1.3 (0.8,2.3)	0.3 (−0.2,1)	<0.001
		*p*	<0.001	0.35	0.78	0.019	0.78	0.89	0.62	0.61	0.006	0.77	0.27	<0.001	

**Table 3:** Statistical analysis of the intra-group PTV differences between the teeth demonstrating higher and lower stability.

	Examination I				Examination II				Examination III			
Group	M	S	M	S	M	S	M	S	M	S	M	S
Stability	Median (IQR)		Mean		Median (IQR)		Mean		Median (IQR)		Mean	
Higher +C+, +M1+	0 (−0.2,0.2)	0 (−0.3,0.2)	−0.05	−0.07	0.1 (−0.1,0.5)	0 (−0.2,0.2)	0.15	−0.07	0.5 (0,1.15)	0 (−0.4,0.4)	0.74	−0.07
Lower +I1+, +I2+, +P1+, +P2+	1.45 (0.9,2.6)	1.5 (0.9,2.6)	1.80	1.83	1.5 (1,2.6)	−0.1 (−0.4,0.3)	1.85	1.83	1.55 (1.05,2.75)	1.6 (0.9,2.6)	1.92	1.84
*p*	<0.001	<0.001			<0.001	<0.001			<0.001	<0.001		

Legend: +M1+—the first molars; +P2+—the second premolars; +P1+—the first premolars; +C+—the canines; +I2+—the lateral incisors; +I1+—the central incisors.

**Table 4:** Statistical analysis of both the intra- and the intergroup median PTV differences between all examinations.

	Group M	Group S	Total	*p*
Examination I	1 (0.15,2.1)	1 (0.2,2)	1 (0.2,2.05)	0.667
Examination II	1 (0.2,2.1)	0.9 (0.2,2.1)	1 (0.2,2.1)	0.386
Examination III	1.2 (0.5,2.3)	0.9 (0.1,2.2)	1.1 (0.3,2.2)	<0.001
*p*	<0.001	0.758		

**Table 5:** Statistical analysis of the intergroup PTV differences in examination III.

Tooth	Group M		Group S		
	Median (IQR)	Mean	Median (IQR)	Mean	*p*
+I1+	2.2 (1.3,2.8)	2.35	1.95 (1.4,3.2)	2.21	0.65
+I2+	2.15 (1.5,3.1)	2.48	2.2 (1.2,3)	2.32	0.59
+C+	0 (0,0.2)	0.1	−0.2 (−0.6,0.2)	−0.24	<0.001
+P1+	1.05 (0.4,3)	1.42	1.05 (0.4,2.4)	1.47	0.67
+P2+	1.1 (0.6,2.2)	1.43	1.2 (0.7,2.1)	1.40	0.98
+M1+	1.1 (0.6,1.9)	1.34	0.1 (−0,3,0.6)	0.08	<0.001

Legend: +M1+—the first molars; +P2+—the second premolars; +P1+—the first premolars; +C+—the canines; +I2+—the lateral incisors; +I1+—the central incisors.

## Data Availability

Not applicable.
